# Long non-coding RNA PVT1 indicates a poor prognosis of glioma and promotes cell proliferation and invasion via target EZH2

**DOI:** 10.1042/BSR20170871

**Published:** 2017-12-15

**Authors:** Anqiang Yang, Handong Wang, Xiaobing Yang

**Affiliations:** 1 Department of Neurosurgery, School of Medicine, Jinling Hospital, Southern Medical University (Guangzhou), 305 East Zhongshan Road, Nanjing 210002, China; 2 Department of Neurosurgery, The First People’s Hospital of Yibin City, 65 Wenxing Road, Yibin 644000, China

**Keywords:** EZH2, glioma, invasion, PVT1, proliferation

## Abstract

Human glioma is one of the malignant tumors of the central nervous system (CNS). Its prognosis is poor, which is due to its genetic heterogeneity and our poor understanding of its underlying molecular mechanisms. The present study aimed to assess the relationship between plasmacytoma variant translocation 1 (PVT1) and enhancer of zeste homolog 2 (EZH2), and their effects on the proliferation and invasion of glioma cells. The expression levels of PVT1 and EZH2 in human glioma tissues and cell lines were measured using quantitative RT-PCR (qRT-PCR). Then, after siRNA-PVT1 and entire PVT1 sequence vector transfection, we determined the regulation roles of PVT1 in the proliferation, apoptosis, migration, and invasion of glioma cells. We found that the expression levels of both PVT1 and EZH2 were up-regulated in human glioma tissues and cell lines, and positively correlated with glioma malignancy. And, silencing of PVT1 expression resulted in decreased proliferation, increased apoptosis, and decreased migration and invasion. In addition, exogenous PVT1 led to increased EZH2 expression and increased proliferation and induced proliferation and invasion. These data inferred that long non-coding RNA PVT1 could be served as an indicator of glioma prognosis, and PVT1–EZH2 regulatory pathway may be a novel therapeutic target for treating glioma.

## Introduction

Glioma, a malignant tumor of the central nervous system (CNS), accounts for 70% of all brain tumors each year [1]. The patient survival rates strongly depend on the histological grades of the tumors. Although its progress has been retarded in surgery, radiotherapy, and chemotherapy, the prognosis of patients with glioma is still poor due to its rapid and invasive growth, its genetic heterogeneity, and our poor understanding of its underlying molecular mechanisms [2,3].

Long non-coding RNAs (lncRNAs), which are more than 200 bases in length and unable to be translated into proteins, have been demonstrated to play crucial roles in tumorigenesis in recent years [4]. Emerging evidence suggests that lncRNAs may play critical roles in cellular development, differentiation, and many other biological processes [5]. The oncogenic effects of PVT1 have been further highlighted by more recent studies demonstrating its overexpression and amplification in multiple cancer types [6,7]. Recent study found that PVT1 contributed to tumorigenesis of thyroid cancer through recruiting enhancer of zeste homolog 2 (EZH2) [8], and promoting glioma vascular endothelial cell proliferation, migration, and angiogenesis by targetting *miR-186* [9]. More importantly, PVT1 expression has been significantly correlated with clinical features such as recurrence and survival in various cancers [10,11]. However, the functional role and molecular mechanism of PVT1 in glioma remain unclear.

In the present study, first, we had measured the aberrant PVT1 and EZH2 expression in clinical glioma tissue samples and glioma cell lines. Then, we investigated the potential effects of PVT1 on glioma cell proliferation and invasion using two kinds of glioma cell lines. siRNA-mediated gene silencing and entire PVT1 gene vector mediated gene overexpression were respectively used to assess the effects of lncRNA PVT1 on cell proliferation and invasion *in vitro*, and tumor formation *in vivo*. The present study aimed to investigate the potential roles of PVT1 in the development of glioma and to elucidate its underlying mechanism.

## Materials and methods

### Cell culture and tissue sample collection

The normal glial cell line HEB and glioma cell lines U87MG and U251 were purchased from the Shanghai Cell Collection (Shanghai, China). The cells were cultured in Dulbecco’s modified Eagle’s medium (DMEM) supplemented with 10% FBS at 37°C in a 5% CO_2_ humidified atmosphere. Eighty clinical glioma tissue samples and ten normal brain tissue samples came from Jinling hospital. All samples were histopathologically examined at the Jinling hospital (Guangzhou, China) and, according to the World Health Organization (WHO) criteria, classified into four grades: primary grade pilocytic astrocytoma (WHO I), grade II astrocytoma (WHO II), grade III anaplastic astrocytoma (WHO III), and grade IV glioblastoma multiforme (GBM; WHO IV). Of each grade 20 cases were included.

### Quantitative RT-PCR assay

Total RNA was extracted from the tissue and cells by using TRIzol reagent (Invitrogen, U.S.A.). The cDNA Synthesis Kit (Takara, China) was used for the synthesis of cDNA according to the manufacturer’s instructions. Quantitative RT-PCR (qRT-PCR) was performed to detect the expression levels of *PVT1* and *EZH2* mRNA. *β-actin* mRNA levels were used for normalization. The qRT-PCR results were analyzed and expressed as relative mRNA levels of the *C*_T_ value, which was then converted into fold-change.

### Synthesis and transfection of interfering RNA

The entire PVT1 sequence was amplified with RT-PCR and then cloned into the expression vector pCDH-MSCV-EF1-GFP-T2A-Pu (System Biosciences). siRNA-PVT1 (sense: 5′-UUAGUAUCCUGAAAUGUGC-3′, antisense 5′-GCACAUUUCAGGAUACUAA-3′) and its control (sense: 5′-UUCUCCGAACGUGUCACGUTT-3′, antisense: 5′-ACGUGACACGUUCGGAGAATT-3′) were synthesized and purchased from GenePharma (Shanghai, China). Twenty-four hours prior to transfection, cells were plated on to a six-well plate at 60–70% confluence. Transfection was performed using Lipofectamine 2000 (Invitrogen, U.S.A.) according to the manufacturer’s protocol. The medium was replaced 4–6 h after transfection with new complete medium. After transfection into U87MG and U251 cells, positive clones were selected using 1 μg/ml puromycin and propagated further.

### Flow cytometry analysis of apoptosis rates

Apoptosis was determined using the apoptosis detection kit according to the manufacturer’s instructions. Briefly, cells were collected and washed twice in PBS and resuspended at a density of 1 × 10^3^ cells/ml. Next, the transfected cells were simultaneously incubated in the dark with PE-labeled Annexin V and 7-ADD for 20 min and analyzed using a flow cytometer (BD, Accuri C6, CA, U.S.A.).

### Flow cytometry analysis of cell cycle and proliferation index

Analysis of the distribution of U87MG and U251 cells along the G_0_/G_1_, S, and G_2_/M phases of cell cycle was performed after transfection. The cells were harvested, suspended in PBS, and fixed with 70% ethanol at 4°C overnight. After washing the cells twice in ice-cold PBS and resuspending in binding buffer, the cellular DNA was stained with 5 μl propidium iodide (BD, U.S.A.) and samples were analyzed by the FACS Calibur flow cytometer (BD, Accuri C6, U.S.A.). Proliferation index (PI) was calculated as per the following formula: PI = (S + G_2_/M)/(G_0_/G_1_ + S + G_2_/M).

### Transwell migration assay

Migration assay was performed using 6.5-mm transwell inserts with polycarbonate membrane filters containing 8-mm pores (Corning, U.S.A.). Approximately 1 × 10^5^ cells resuspended in 200 μl serum-free medium were plated in the upper transwell chamber. Six-hundred microliters of medium with 10% FBS was added to the lower well. Following incubation for 48 h, cells that remained on the top of the filter were removed, and cells that migrated to the lower surface were fixed in 4% paraformaldehyde followed by staining with 0.1% Crystal Violet dye. Five fields were counted randomly to calculate the number of cells invading through the polycarbonate membrane (100×). Each experiment was performed in triplicate.

### Matrigel invasion assay

Invasion assay was performed using 6.5-mm transwell inserts with polycarbonate membrane filters containing 8-mm pores (Corning, U.S.A.) followed by incubation of matrigel on top of the membranes for gel formation. The following steps were the same as those for the transwell migration assay.

### Tumor formation experiment

Female nude mice at the age of 6–8 weeks were randomly divided into four groups (five mice per group): silence-NC group, silence group, overexpression-NC (Over-NC) group, and overexpression (Over) group. The U251 cell suspension (2 × 10^7^ cells/ml) in 200 μl serum-free medium was subcutaneously injected into the flanks of the mice, respectively. During the following 4 weeks, the tumors were carefully observed and measured. To the end, fresh tumors were weighted, measured, and used for the detection of Western blot assay.

### Western blot assays

Fifty microgram per lane of total cell lysates was resolved on SDS/PAGE gels, followed by immunoblot detection and visualization with ECL Western blot detection reagents (Pierce Biotechnology, U.S.A.). Immunoblotting was performed with appropriate primary antibodies. The protein bands were scanned and quantitated as relative band intensity.

### Statistical methods

SPSS17.0 statistical software was used for statistical analysis. The significance was determined with the Student’s *t* test or one-way ANOVA. The measurement data were represented as mean ± S.E.M. (X ± s). *P*<0.05 indicated statistically significant differences.

## Results

### PVT1 and EZH2 were up-regulated in glioma tissues and cell lines and correlate with poor prognosis

**Figure 1:**
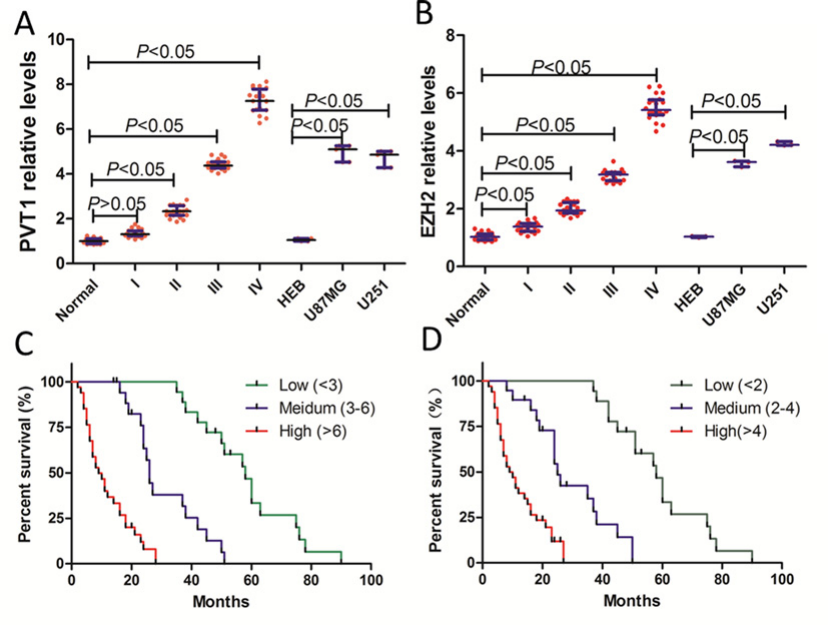
PVT1 and EZH2 were up-regulated both in glioma tissues and cell lines (**A**) qRT-PCR analysis of PVT1 expression in glioma tissues and cell lines. (**B**) qRT-PCR analysis of EZH2 expression in glioma tissues and cell lines. Data are the mean ± S.E.M. (tissue samples *n*=20, cell samples *n*=3). (**C**) Log-rank (Mantel–Cox) of survival curves was analyzed according to PVT1 expression levels. (**D**) Log-rank (Mantel–Cox) of survival curves was analyzed according to EZH2 expression levels.

The expressions of PVT1 and EZH2 in normal brain tissues, glioma tumor tissues, and glioma cell lines were analyzed by qRT-PCR. qRT-PCR analysis demonstrated that PVT1 and EZH2 were expressed at higher levels in glioma tumor tissues and cell lines than normal brain tissues and HEB cells, and positively correlated with glioma malignancy (glioma grades, Figure 1A,B), which mean the higher the malignant grade of glioma, the higher the PVT1 and EZH2 expression level.

Moreover, to explore the relationship between PVT1 expression and glioma patients’ prognosis, we attempted to assess the correlation between PVT1 levels and clinical outcomes. First, we divided the patients into three groups based on *PVT1* mRNA expression levels (low: <3, *n*=18; medium: 3–6, *n*=19; high: >6, *n*=34) and then conducted Kaplan–Meier analysis. The results (Figure 1C) showed that the median survival time for high PVT1 expression was 9.5 months, while it was 25 months for medium PVT1 expression, and 58 months for low PVT1 expression.

In addition, we also assessed the correlation between EZH2 levels and clinical outcomes. Similarly, we divided the patients into three groups based on *EZH2* mRNA expression levels (low: <2, *n*=16; medium: 2–4, *n*=17; high: >4, *n*=38) and then conducted Kaplan–Meier analysis. The survival curves (Figure 1D) showed that the median survival time for high EZH2 expression was 10.4 months, while it was 28 months for medium EZH2 expression, and 57.6 months for low EZH2 expression.

Taken together, these results suggested that both PVT1 and EZH2 expression levels were positively correlated with glioma malignancy, and may play an important role in glioma progression.

### PVT1 regulates EZH2 expression levels

**Figure 2:**
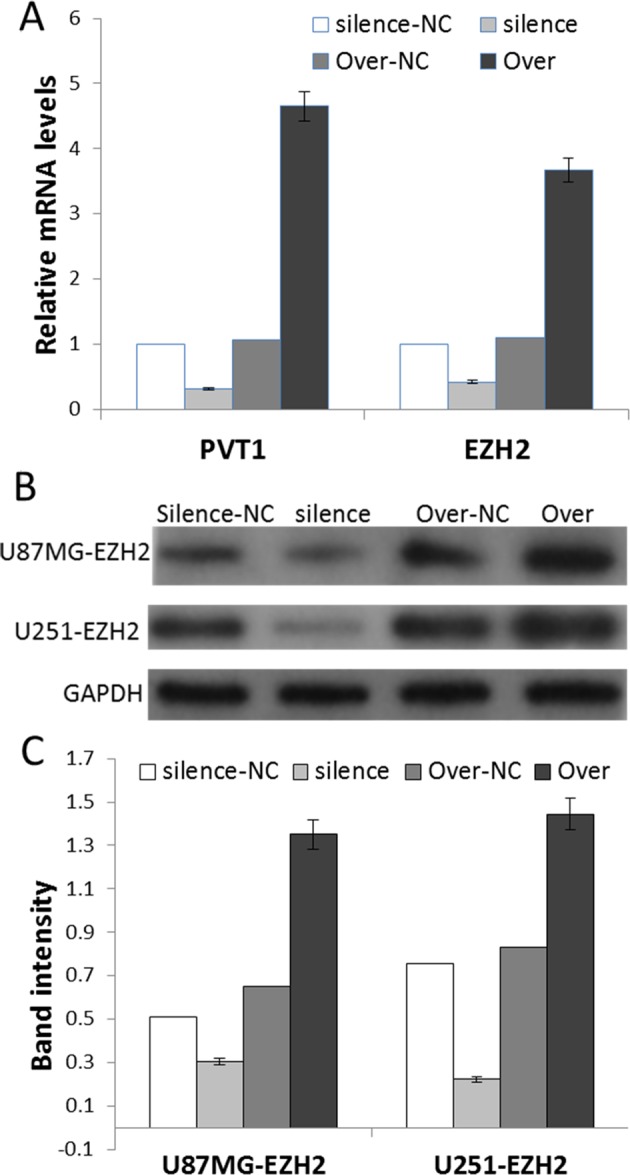
PVT1 regulates EZH2 expression levels *in vitro* (**A**) The expression of *EZH2* mRNA in U87MG and U251 cells after transfection with siRNA-PVT1 or entire PVT1 sequence vector by qRT-PCR. (**B**) Western blot analyses of EZH2 proteins in U87MG and U251 cells in each group. (**C**). Gray scale analyses of the relative EZH2 expression levels. The data are presented as mean ± S.E.M. (*n*=3). Silence group compared with silence-NC group, *P*<0.05; Over group compared with Over-NC group, *P*<0.05.

After silence and overexpression PVT1 transfection, qRT-PCR was employed to detect the transfection efficiency and EZH2 expression levels in U87MG and U251 cells. As shown in Figure 2A, the expression of EZH2 was inhibited after transfection with siRNA-PVT1 (silence-NC and silence groups), and was promoted after transfection with the entire PVT1 sequence vector (Over-NC and Over groups). There was statistical difference between these NC and transfected groups (*P*<0.05). Furthermore, the effects of PVT1 expression on endogenous EZH2 protein were monitored by Western blot. It showed that siRNA-PVT1 can inhibit EZH2 protein expression, and the entire PVT1 sequence vector can induce EZH2 protein expression (Figure 2B,C, *P*<0.05).

### Silence and overexpression of PVT1 regulate the apoptosis and cell cycle

**Figure 3:**
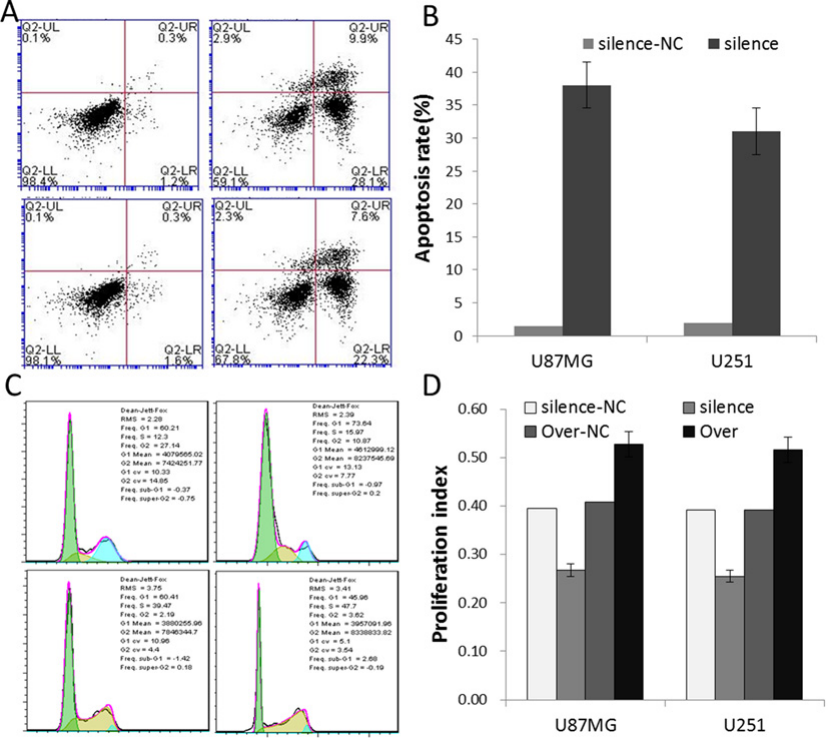
Silence and overexpression of PVT1 affect the apoptosis and cell cycle Flow cytometry analysis of the apoptosis and cell cycle. (**A**) Macroscopic appearance of apoptosis in the silence-transfected groups. There was no significant apoptosis in the overexpression-transfected groups (which were not shown). (**B**) The statistical results of apoptosis rates in the silence-transfected groups. (**C**) Macroscopic appearance of cell cycle in U87MG cells. (**D**) The statistical results of PI in U87MG and U251 cells. PI = (S + G_2_/M)/(G_0_/G_1_ + S + G_2_/M). Data are the mean ± S.E.M. (*n*=3). Silence group compared with silence-NC group, *P*<0.05; Over group compared with Over-NC group, *P*<0.05.

To explore the role of PVT1 in glioma cells, we stably silenced PVT1 with siRNA-PVT1, and overexpressed PVT1 with entire PVT1 sequence vector respectively in U87MG and U251 cells. Flow cytometric analysis of apoptosis and cell cycle showed that silencing of PVT1 inhibited cell proliferation (PI decreased) and induced apoptosis (apoptosis rates increased), and overexpression of PVT1 promoted cell proliferation (PI increased) and had no significant effect on apoptosis (Figure 3). These results suggested that PVT1 regulates glioma cell apoptosis and proliferation.

### Silence and overexpression of PVT1 regulate glioma cell migration and invasion *in vitro*

**Figure 4:**
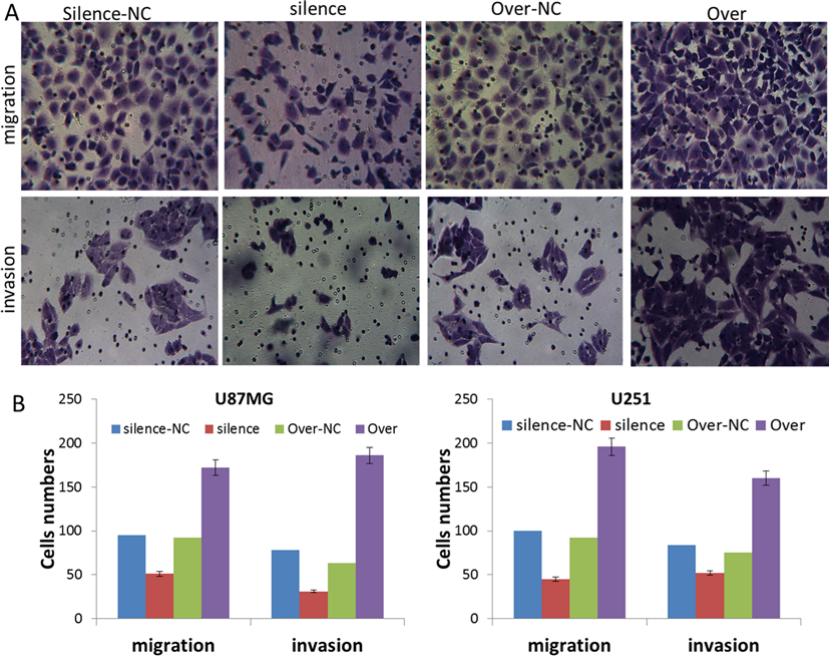
Silence and overexpression of PVT1 regulate glioma cell migration and invasion *in vitro* (**A**) Migration and invasion assay after silence and overexpression transfection, respectively. Representative photographs were taken at 200× magnification. (**B**) The statistical results of migrated and invaded cell numbers in U87MG and U251 cells in each group. Silence group compared with silence-NC group, *P*<0.05; Over group compared with Over-NC group, *P*<0.05.

As shown in Figure 4A,B, transwell assay showed that the migration and invasion capacity were markedly reduced in silence group, showing the decrease in the number of migrated and invaded cells, and that the migration and invasion capacity were markedly promoted in Over group, showing significant increase in the number of migrated and invaded cells. There was statistical difference between these NC and transfected groups (*P*<0.05). These results suggest that silencing of PVT1 reduces the abilities of migration and invasion, and overexpression of PVT1 induces the abilities of migration and invasion of U87MG and U251 cells.

### Silence and overexpression of PVT1 regulate tumorigenesis *in vivo*

**Figure 5:**
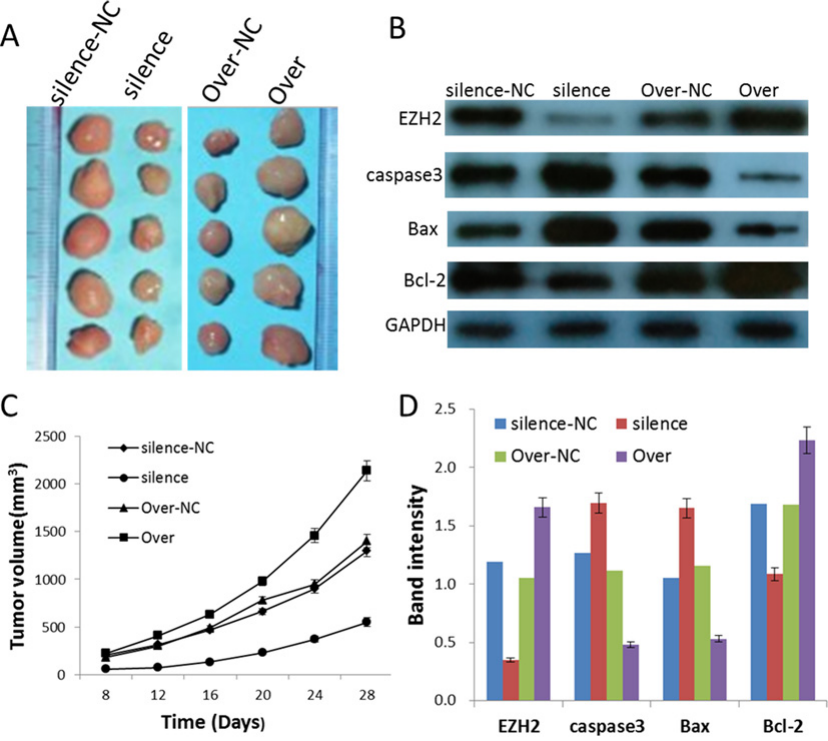
Silence and overexpression of PVT1 regulate tumorigenesis *in vivo* (**A**) Photographs of tumors excised 28 days after inoculation of stably transfected U251 cells into nude mice. (**B**) Western blot analyses of apoptosis-related proteins (caspase 3, Bax, and Bcl-2). (**C**) Mean tumor volume measured by caliper during the experiment. (**D**) Gray scale analyses of the relative EZH2, caspase 3, Bax, and Bcl-2 expression levels. The data are presented as mean ± S.E.M. (*n*=5). Silence group compared with silence-NC group, *P*<0.05; Over group compared with Over-NC group, *P*<0.05.

To study the effect of PVT1 on the tumorigenesis, U251 cells transfected with the siRNA-PVT1 and entire PVT1 sequence vector respectively were used in the nude mice xenograft model. Up to 28 days, there was a dramatic decrease in tumor volume in the silence group compared with silence-NC group, and a marked increase in the Over group compared with Over-NC group (Figure 5A,C). In addition, Western blot analyses of apoptosis-related proteins indicated: when EZH2 expression decreased, the expression of proapoptotic proteins caspase 3 and Bax in silence group increased and the expression of anti-apoptotic protein Bcl-2 decreased. While the expression trends of caspase 3, Bax, and Bcl-2 were just the opposite (Figure 5B,D). Combined with the previous research, these results suggest that the level of PVT1 expression significantly regulates the tumorigenesis of glioma cells *in vivo*.

## Discussion

The current study revealed that PVT1 expression levels in glioma were significantly higher than in normal brain tissue and that the high PVT1 expression levels correlated with patients’ poor overall survival. Actually, aberrant expression of many lncRNAs has been proved to be the prognostic indicators [12]. For example, HOTAIR up-regulation was correlated with non-small-cell lung cancer advanced pathological stage and lymph node metastasis, and patients with high levels of HOTAIR expression had a relatively poor prognosis [13]. Multivariate analysis showed that the increased lncRNA MALAT1 expression was an independent poor prognostic factor for pancreatic patients [14]. In addition, Ding et al. [15] reported that PVT1 was a new biomarker for human gastric cancer and may indicate lymph node invasion. In the present study, the PVT1 expression levels of four glioma grades showed that it was positively correlated with glioma malignancy, and may play an important role in glioma progression, and may be useful as a prognostic indicator for malignant glioma.

Many previous references have reported the physiological and pathological roles of PVT1, such as in the diabetic nephropathy [16] and colorectal cancer [17]. However, the underlying role and molecular mechanism of PVT1 in glioma remain unclear. The present study’s results showed that PVT1 knockdown could significantly inhibit glioma cell proliferation and invasion, and PVT1 overexpression could conversely induce cell proliferation and invasion both *in vitro* and *in vivo*. These results showed that PVT1 could regulate glioma cell progression, and the molecular mechanism should be researched in depth.

Previous studies have proved that lncRNAs are the key regulators in gene expression. Dysregulated lncRNAs are involved in many pathological processes through miRNAs and target proteins [18]. Shen et al. [19] indicated that PVT1 directly targets *miR-195* and could decrease *miR-195* expression via enhancing histone H3K27me3 in cervical cancer. PVT1 modulated thyroid cancer cell proliferation by recruiting EZH2 and regulating thyroid-stimulating hormone receptor [8]. Overexpression of lncRNA PVT1 in gastric carcinoma promoted the development of multidrug resistance and influenced the expression of MDR-related proteins (MDR1, MRP1, mTOR, and HIF-1a) [20]. In addition, Takahashi et al. [11] found that PVT-1 could activate TGF-β signaling pathway and apoptotic signals, resulting in promoting apoptosis in colorectal cancer cells. PVT1 recruited EZH2 to the large tumor suppressor kinase 2 (LATS2) promoter and repressed LATS2 transcription, and PVT1/EZH2/LATS2 interactions might serve as new target for lung adenocarcinoma diagnosis and therapy [21]. In our study, we found that EZH2 expression levels were positively correlated with glioma malignancy and with poor prognosis in glioma tissue samples, which is the same as for PVT1. So, we doubted whether there was a relationship between them. To verify this hypothesis, we measured the EZH2 expression after PVT1 transfection, and found that PVT1 knockdown could down-regulate EZH2 expression and PVT1 overexpression could up-regulate *EZH2* mRNA and protein levels *in vitro* and *in vivo*. Therefore, we infer that PVT1 may modulate glioma cell proliferation and invasion via EZH2.

EZH2 is the human homolog of the *Drosophila* protein Enhancer of Zeste (E(z))2 and contains a SET domain that often modulates cell growth pathways [22,23]. Overexpression of EZH2 is a marker of advanced and metastatic disease in many solid tumors, including prostate and breast cancer [24]. In addition, EZH2 was more expressed in GBM than in low-grade glioma, and inhibition of EZH2 expression by shRNA could decrease glioma cell proliferation [25], and high expression of EZH2 was determined to be an independent predictor of short overall survival [26]. Our study also found that the EZH2 is high expression in malignant glioma cells, which might be a predictor of overall survival. Moreover, we inferred that PVT1 was an upstream regulatory gene of EZH2, and PVT1 may modulate glioma cell proliferation and invasion via EZH2. However, the exact regulatory mechanism in glioma progression, including whether it involves miRNAs, requires further study.

In conclusion, the identification of PVT1 as an important prognostic factor for glioma patients induced our interests to explore its functional roles, and finally we found that PVT1 could regulate glioma cell proliferation and invasion both *in vitro* and *in vivo*. Importantly, we first found that PVT1 may regulate glioma proliferation and invasion via target EZH2. The present study may provide a strategy and facilitate the development of lncRNA-directed diagnostics and therapeutics against glioma.

## Abbreviations

Bax: Bcl-2 associated X protein
Bcl-2: B-cell lymphoma-2
CT: Cycle threshold
EZH2: enhancer of zeste homolog 2
GBM: glioblastoma multiforme
lncRNA: long non-coding RNA
LATS2: large tumor suppressor kinase 2
Over: overexpression group
Over-NC: overexpression-NC group
PE: phycoerythrin
PI: proliferation index
PVT1: plasmacytoma variant translocation 1
qRT-PCR: quantitative RT-PCR
TGF-β: Transforming growth factor-β
WHO: World Health Organization
7-ADD: 7-amino-actinomycin D
